# A study of sheep scab in Northern Ireland including detection and identifying barriers to control

**DOI:** 10.1002/vro2.70003

**Published:** 2024-12-23

**Authors:** Paul Crawford, Sharon Verner, Sam Strain, Adewale Henry Adenuga, Aurélie Aubry, Stewart Burgess

**Affiliations:** ^1^ Northern Ireland Sheep Scab Group Islandmagee UK; ^2^ Animal Health and Welfare Northern Ireland Dungannon UK; ^3^ Economics Research Branch Agri‐Food and Biosciences Institute Belfast UK; ^4^ Sustainable Livestock Systems Branch Agri‐Food and Biosciences Institute Hillsbourgh UK; ^5^ Moredun Research Institute Pentlands Science Park Midlothian Scotland UK

## Abstract

**Background:**

Sheep scab, caused by the highly infectious *Psoroptes ovis* mite, is considered to be endemic in Northern Ireland, although little investigation has been reported. A pilot project was undertaken to engage farmers, confirm cases with diagnostic methods and identify specific barriers to control, with the aim of informing future control programmes.

**Methods:**

Through farmers self‐reporting suspected outbreaks, on‐farm risk assessments and clinical investigations were carried out by the farm's veterinary surgeon, who utilised light microscopy and serological testing to diagnose scab. Treatment was then provided and where macrocyclic lactones (MLs) were utilised, follow‐up testing was attempted.

**Results:**

Sheep scab was identified in 60 flocks across all six counties of Northern Ireland. Serological testing proved essential in uncovering scab infestation where light microscopy failed to identify mites, or where no suitable lesions existed to scrape. Where MLs were used, follow‐up was incomplete. Furthermore, four of six resampled flocks still showed a positive result. Barriers to better scab control included poor quarantine arrangements and preventative treatment strategies that ultimately proved ineffective.

**Conclusions:**

The project demonstrated that farmers were willing to engage in control efforts, they appreciated the support provided in managing outbreaks and they recognised the need for a coordinated effort to control scab. Greater awareness of biosecurity is needed among farmers. Facilitation of farmer‐driven scab control activities is urgently needed, alongside greater understanding of the scale of the disease and the impact that ML treatment failure can have on scab dissemination through the national Northern Ireland flock.

## INTRODUCTION

Sheep scab, caused by an allergic reaction to a faecal antigen produced by the highly infectious, surface‐living mite *Psoroptes ovis*,^1^, ^2^ is a notifiable disease in Northern Ireland.^3^ A survey in Northern Ireland highlighted significant knowledge gaps among respondents and evidence of poor practice in diagnosis, prevention and treatment^4^; furthermore, farmers self‐reported outbreaks with a frequency at least five times higher than state figures for the same period. Farmers reported, almost exclusively, a reliance on visual assessment of sheep to identify potential scab incursions.

Prior work has demonstrated that a cornerstone of scab control is rapid and accurate diagnosis of infestation to limit local spread, as well as appropriate biosecurity, in particular, when sheep are moved from one area to another. Skin scrapes can provide a rapid, potentially pen‐side and sensitive diagnosis of sheep scab; however, failure to detect mites is well recognised.^5^ A blood test (ELISA) developed to detect antibodies specific for the scab mite allergen, Pso o 2, has provided high levels of sensitivity and specificity for the detection of sheep scab and is capable of detecting infestation before the appearance of clinical signs.^1^, ^6^ A blood test approach has been demonstrated to reduce the number of unnecessary treatments needed among ‘at‐risk’ sheep in a disease cluster scenario^6^, ^7^ by accurately identifying *P. ovis‐*infested flocks.

Founded after an open meeting for the sheep sector in 2019, the Northern Ireland Sheep Scab Group, a farmer‐driven, industry‐wide partnership focused on developing plans for improved scab control in Northern Ireland.^9^, ^10^ A partnership was developed with the Moredun Research Institute, Agri‐Food and Biosciences Institute and Animal Health and Welfare, Northern Ireland. Funding was obtained from BBSRC's Endemic Livestock Diseases programme to undertake pilot research into sheep scab in Northern Ireland. This was the first study in Northern Ireland and aimed to establish a self‐reporting scheme for farmers concerned about potential scab incursions into their flock, as well as a field study to determine if these cases were caused by *P. ovis*, providing support in treatment where it was diagnosed. In parallel, a range of other activities to explore knowledge gaps, including a survey to consider the economic and environmental consequences of scab in Northern Ireland, were undertaken and reported elsewhere.^11^ Here, we report the data from a field study, which aimed to understand the distribution of sheep scab in Northern Ireland and to identify factors that may be a barrier to scab control.

## METHODS

A helpline was opened on 1 September 2022 for farmers to self‐report suspected scab outbreaks in their flocks. This followed five open discussion group meetings for farmers to explain the scheme, discuss *P. ovis* biology and the current best practice for the control of sheep scab. A further meeting was held for veterinary surgeons (vets) to standardise the approach they would take during flock visits. No restrictions were placed on recruitment, save that the farmer described signs consistent with scab in their sheep or a recent contact that created a high risk of scab incursion into their flock. Farmers who called the helpline were briefed about the scheme and if they wished to participate, verbal consent was obtained and scheme details, including the notifiable status of scab in Northern Ireland, were confirmed in writing. The farmer's vets then, at a mutually convenient time, examined the flock, undertook diagnostic sampling and, where appropriate, discussed treatment options; documenting farm and flock information on a pre‐prepared risk assessment form (RAF) (see Supporting Information S1).

Blood samples were to be taken at all flock visits, with 12 sheep samples taken from each flock or affected management group. Where visible skin lesions, which were typical of sheep scab, were identified, vets were encouraged to take skin scrape samples from the edge of lesion(s) and examine them microscopically for the presence of live *P. ovis*. The taking of skin scrapes was encouraged to shorten the time to diagnosis and treatment where there was a realistic opportunity for the vets to detect mites, with the blood samples taken in parallel as a back‐up (since skin scrapes were not reviewed until the vet returned to their practice) and as a reference point for investigation of potential failure of treatment.

Blood samples were submitted, by post, for analysis at a commercial laboratory. The results were interpreted by the project team. A flock was considered positive based on serology if one or more individual samples exceeded the ELISA optical density (OD) cut‐off value (>50 being positive). Flocks with borderline/suspicious results (ODs between 40 and 50) but with no clinical evidence of, and low risk of, disease were reported as suspicious, or monitor, with the option of follow‐up testing after 4 weeks. High‐risk flocks, such as those with access to common grazing where scab had been confirmed among sheep grazing that common, were offered treatment, even if their flock test showed a negative result.

Medicines for the treatment of flocks were provided by the project, up to a financial cap equivalent to one can of sheep dip concentrate (approximately £400). Organophosphate (OP) plunge dipping was the preferred treatment option under the project; however, where the farmer, in consultation with their vet, identified that an injectable macrocyclic lactone (ML) treatment would be preferable, this was subsequently provided. All farmers using MLs were offered a revisitation by their vet to collect further blood samples to repeat serology to confirm treatment efficacy 4‒6 weeks post‐treatment. This was paid for by the project. While treatment failure following dipping was possible, flocks treated with MLs were targeted for this follow‐up testing because this treatment route was considered to be the highest risk for failure; logistics and budgetary constraints prevented post‐testing treatment of all flocks. Dipping could be undertaken by the farmer if they had the necessary certificate of competency^12^ or by a contract (mobile) dipper.

At the conclusion of all visits, farmers who had consented to participate in the scheme were invited to provide feedback through a short online survey form^13^ (see Supporting Information S2), which was then analysed. Briefly, this consisted of downloading responses into spreadsheets for standard statistical consideration. Questions offering a free‐text response were coded and themes, alongside exemplar quotes, were identified.

## RESULTS

Between 1 September 2022 and 30 June 2023, details of 155 farmers were logged by the helpline, 108 consented to participate and, of these, 105 progressed to a flock visit. No farmers were excluded from participation by the programme following this initial contact. The reasons for attrition included farmers opting to start treatment immediately, rather than waiting for a veterinary assessment, being reluctant to risk being served restriction notices, or taking up the option to move affected animals directly to slaughter. Additionally, a number of callers were not logged because they declined to share any details once informed of the requirement to notify state authorities if scab was detected.

**FIGURE 1:**
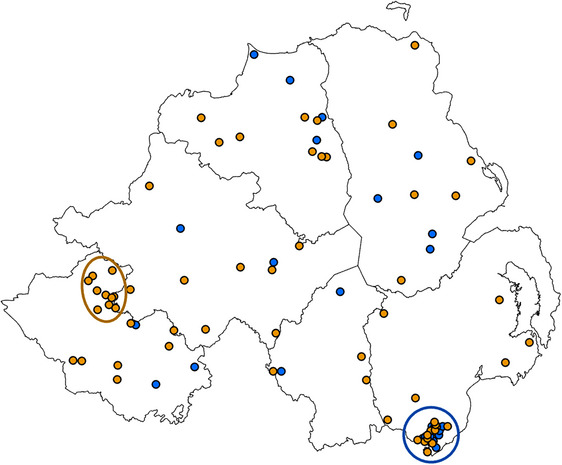
Approximate location of farms that participated in the Northern Ireland sheep scab project, based on a partial postcode of the farm home address, to preserve anonymity, that received a farm visit. Farms postive for sheep scab are indicated with an orange dot and negative flocks with a blue dot. Large, open circles identify two groupings identified during investigations (image created using ArcGIS Pro 3.2.2).

**FIGURE 2:**
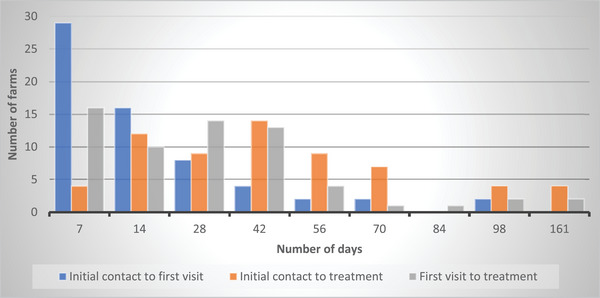
The response time in days for farmers participating in the Northern Ireland sheep scab project to arrange a farm visit from their veterinary surgeon and for treatment to be delivered in flocks returning a positive diagnosis for sheep scab by light microscopy or serology.

The approximate locations of farms participating in the project are shown in Figure 1. The time from initial contact with the project helpline to the flock visit varied, as did the time to treatment (Figure 2). Blood samples from a small number of flocks were delayed in the postal service for more than 14 days due to strike action and two were not suitable for analysis. Other samples were delayed for shorter periods but were suitable for analysis, albeit with a resultant delay to treatment. As notification of flock restriction and de‐restriction were made directly from the state authority to the farmer, scheme managers were not able to accurately determine the timescale for de‐restriction in every case. However, while many restrictions were lifted within 72 h of treatment, delays beyond 14 days were brought to the project's attention by the participating farmers. The following results relate to the initial flock visit and investigation, unless otherwise stated.

### Flock visit risk assessment findings

While data capture was incomplete on many RAFS and, on two occasions, the vet did not return a RAF, a range of issues that could form risks or barriers to optimal scab control on the farms visited were identified.

Farm size (range 1‒283 hectares, mean 53.7, *n* = 87) and flock size (ranging from no breeding ewes [store lamb enterprise only] to 1250 ewes, mean 172, *n* = 100) varied considerably; however, no statistically significant differences were found, using Welch's *t*‐test, between farms with positive and negative sheep scab status.

The responses indicated that, on average, participating flocks had 4.4 contiguous flocks (range 0‒20 flocks, *n* = 97). However, when asked if they knew about any neighbours having had scab recently, only 14 (14%) of 101 farmers responding stated they knew that their neighbours had scab in their flock, with the majority (58, 57%) indicating that they did not know the scab status of their neighbours’ flocks.

The majority (71, 69%) of the farms had not had a previous scab outbreak. Of the 26 (25%) that had outbreaks, the majority (*n* = 18) had occurred within the previous 3 years. A variety of treatments had been used to treat previous outbreaks. Doramectin was used most (18 times, 55% of previous outbreaks). Three farms used one or more injectable MLs followed by diazinon plunge dipping and one farm used doramectin followed by moxidectin.

When asked about the potential source of the scab outbreak, of the 53 flocks diagnosed with scab, 21 respondents (40%) blamed purchased sheep, with six (11%) specifically mentioning markets. Neighbouring flocks (*n* = 19, 36%), straying sheep (*n* = 3, 6%) and common grazing (*n* = 3, 6%) were also mentioned. Five farmers (9%) indicated that they did not know how scab could have entered their flock.

When asked about the source of replacement, sheep most (58, 59%, *n* = 99) bought ewes and 91% (84, *n* = 92) bought rams. While not all farmers indicated the source of these purchases, markets were mentioned by 34% in relation to ewe purchases and 51% for ram purchases. When asked if all bought‐in (or returning) animals were yarded or quarantined upon arrival, a substantial minority (31, 31%, *n* = 101) said no. One farmer, who described their flock as being closed, had a positive scab diagnosis; however, further questioning revealed that he occasionally bought rams.

The use of medicines to prevent sheep scab was reported on 57 RAFs. Among the flocks positive for scab, 40 had used at least one product in the previous 12 months. Doramectin was the most commonly utilised product in both negative (*n* = 8) and positive (*n* = 26) flocks. Shower systems for the application of diazinon were mentioned four times. The incorrect administration of doramectin by the subcutaneous route (rather than intramuscular) was reported once. Specific reference to the incorrect use/application of doramectin as a preventative treatment on part of the flock (while providing no residual protection against sheep scab infestation) was reported twice.

### Outcome of clinical investigations

Blood samples were taken from only 96 flocks, despite the request to blood sample all flocks. No sample was received on nine occasions where the vet detected live mites and samples from two flocks were not analysed due to sample deterioration following strike action delaying postal delivery. Sheep scab was confirmed on 56 farms initially, either by detection of live *P. ovis* mites (28 flocks of 54 skin scraped) and/or by serology (47 flocks of 94 flocks blood samples analysed). No vet reported detecting dead mites only in skin scrapes. An inconclusive serology result was obtained from eight flocks, four of which, on serological retesting, showed a positive result. Therefore, scab was confirmed on 60 unique farms. Among the 25 farms with a negative skin scrape, only five (20%) of these were serologically negative. One skin scrape result was reported by the vet as ‘inconclusive’ from a flock, which showed obvious clinical signs and a positive serology result.

Among the flocks where clinical investigations were undertaken, 29 farmers reported no signs of scab; these investigations were triggered due to an identified risk of *P. ovis* incursion. Shared access to common grazing, where scab‐infested sheep were known to have grazed was the most frequent reason. Despite the lack of any clinical signs detectable by the farmer, seven (24%) showed a positive result on serology and an additional six (21%) were recommended to be re‐tested in 2‒4 weeks because they showed equivocal results. Thus, only 16 (55%) of these visually unaffected flocks were serologically negative.

In subsequent results and discussions, the term positive refers to any flock that did not return an unequivocal negative serological result, unless otherwise specifically stated.

Dip was supplied to 46 flocks for the treatment of diagnosed *P. ovis* incursions (including four where MLs failed to eradicate scab), to 24 flocks at high risk of an incursion to prevent scab and to seven flocks that showed an inconclusive result and elected to get treated rather than awaiting further testing. Only five farmers whose flocks suffered a scab outbreak had the necessary certificate of competency to undertake dipping; the majority of farmers relied on commercial contract dipping services. There were no reports of suspected failure of diazinon (OP) efficacy. Injectable MLs were used in 16 flocks, of which six flocks agreed to be resampled (Table 1). Serology returned a positive result from four of them. Another farmer whose sheep were not resampled, reported dipping their sheep because of continuing clinical signs following treatment with the ML.

**TABLE 1:** Outcomes for 16 farms that used macrocyclic lactones to treat sheep scab outbreaks in the Northern Ireland sheep scab project.

Six flocks resampled		10 flocks not resampled	
Clinical signs and serology positive	Two flocks	Farmer recognised clinical signs persisted and had already dipped sheep	One flock
No clinical signs yet serology positive	Two flocks	Farmer planning on dipping despite no recurrence of clinical signs for added security	Two flocks
No clinical signs and serology negative	Two flocks	Sold all sheep direct to slaughter following treatment	One flock
		Farmer considered sheep cured and not needing re‐blood tested	Four flocks
		Sheep still itching ‘a bit’ but planning on selling ewes immediately (destination not specified) but retaining lambs	One flock
		Did not answer telephone call	One flock

**FIGURE 3:**
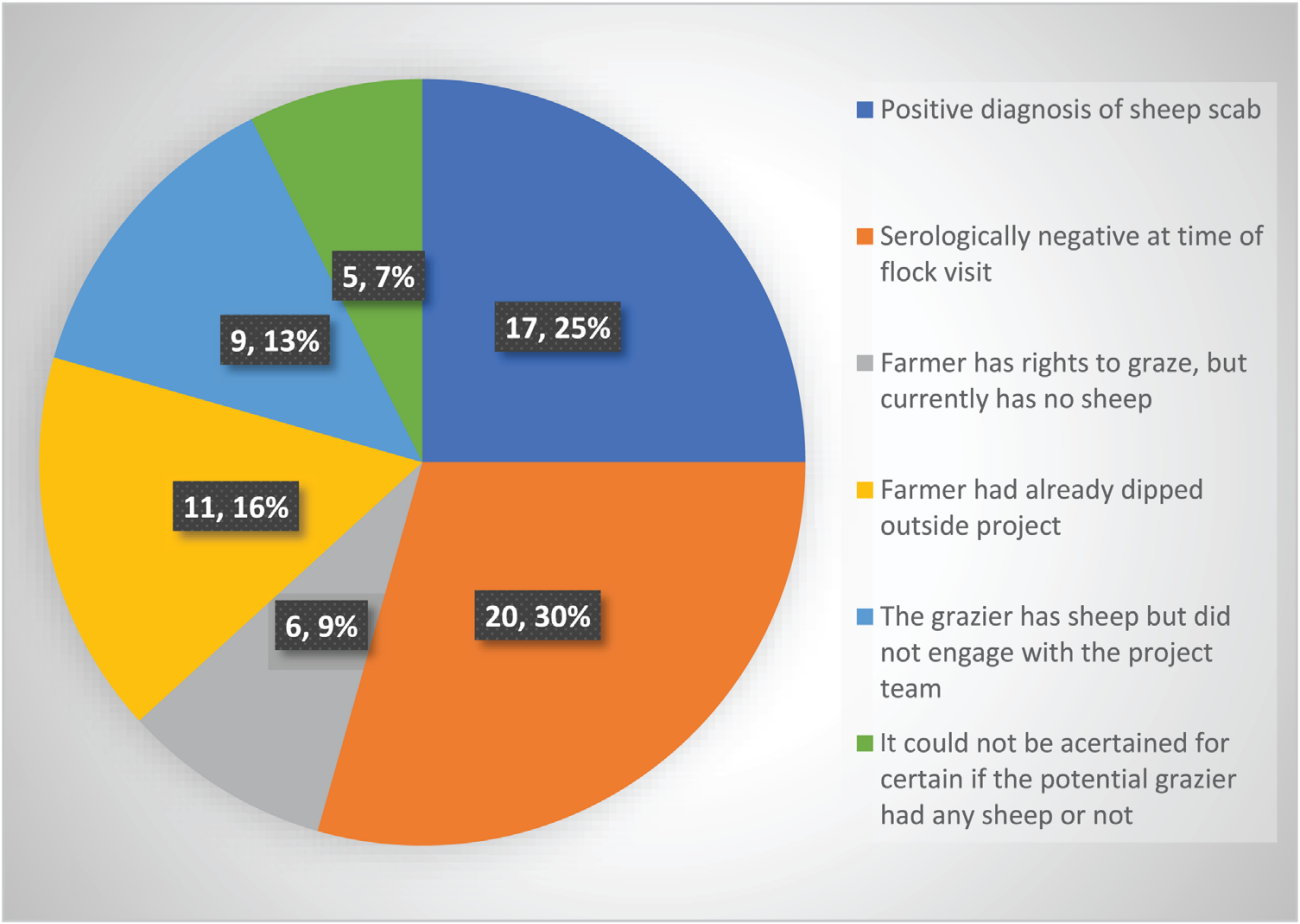
Outcome of attempts to engage the 68 potential graziers on a specific common where sheep scab was identified with the Northern Ireland sheep scab project (number of farms, followed by corresponding percentage).

Two groupings of cases could be seen in the results (Figure 1). One (orange circle) was linked to a proactive vet. Despite offering diagnosis and treatment for free under the project, he reported that he was unable to engage all farmers where he suspected a scab outbreak. The second (blue circle) focused on an area of common grazing where scab had initially been identified in some flocks and then all graziers were encouraged to participate in the project by the common's trustees. However, they could not provide a definitive list of farmers actively grazing sheep on the common. Sixty‐eight names were identified as potentially having rights to graze the common. Of these, 37 participated in the project (Figure 3). Scab was identified, by serology, in sheep belonging to 17 of the graziers. During consultations with their vets, five common graziers were adamant that there was no scab in their flock. Three of these flocks subsequently returned a positive serology result and one was advised to monitor their flock due to an equivocal result.

### Follow‐up survey

**FIGURE 4:**
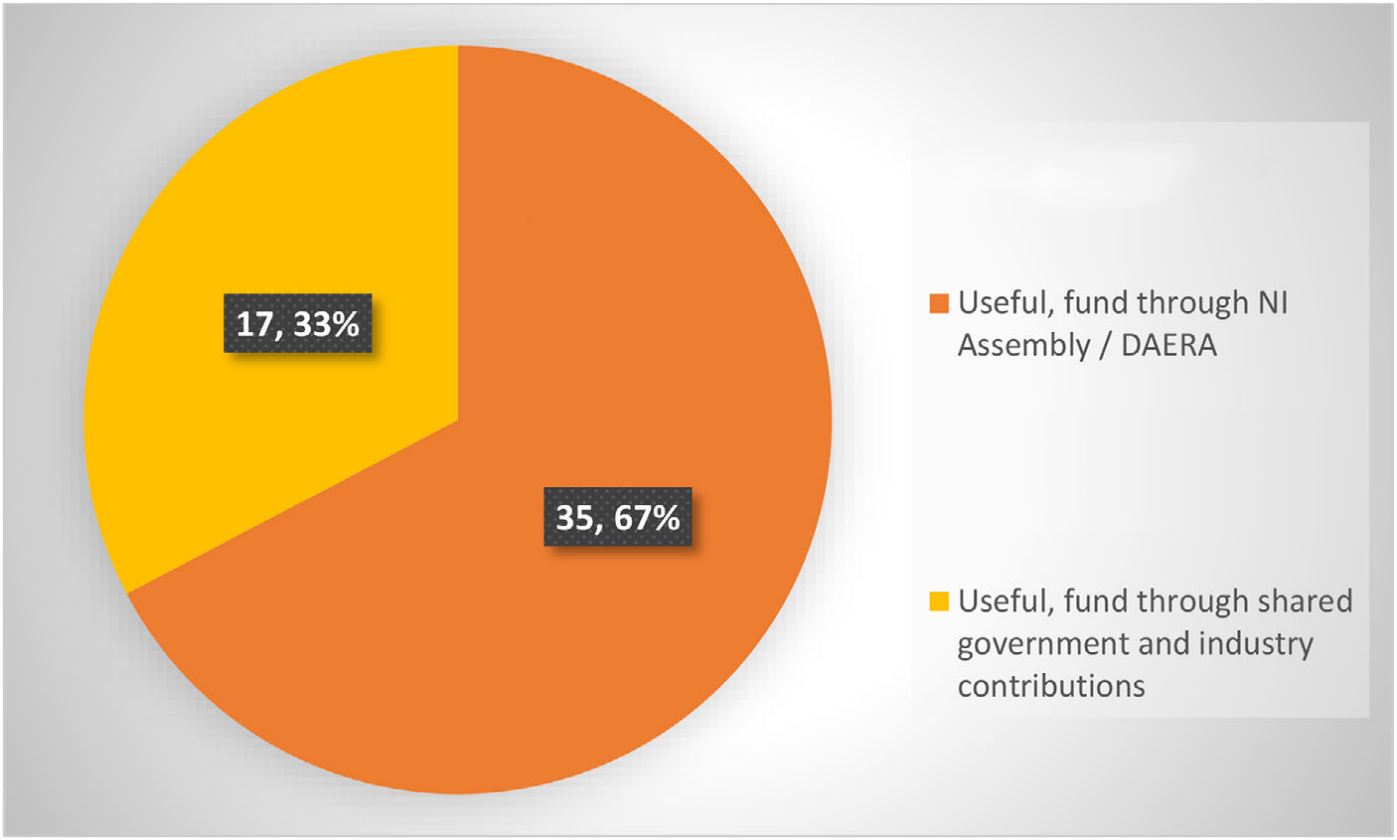
Distribution of responses from 52 farmers who participated in the Northern Ireland sheep scab project to the question ‘Do you think that a programme dedicated to the control of sheep scab in Northern Ireland would be useful in the future and if so, how should this be funded?’ asked as part of follow‐up survey at the project's conclusion (number of farms, followed by corresponding percentage). There were four responses available, no responses were received for the two responses “Not useful” and “Useful, funded through industry contributions”.

Fifty‐two farmers (48% of those invited to participate) responded to the survey. The majority (*n* = 42, 81%) indicated that they would be willing to coordinate future scab treatments with their neighbours and were unanimous in calling for a dedicated sheep scab control programme in Northern Ireland (Figure 4). The participants revealed a wide range of strategies to prevent scab from entering their flock with purchased stock.

The participating farmers made calls for the re‐introduction of compulsory dipping alongside enhanced state controls, such as preventing scab‐infested sheep from moving through markets and wider surveillance to detect scab. The participants also called for further training, increased awareness and the expansion of this project to the national level. Detailed quotes are available in Supporting Information S3.

The project's logistical elements that were welcomed by the participants included having a central contact point, with non‐judgemental people to provide advice. Having their own vet on the farm to undertake the diagnostic testing, paid for by the project, was also well received. Concerns raised focused on delays in getting serology results returned in a timely fashion, getting their flocks de‐restricted; also uncertainty as to whether neighbouring farms and co‐commoners knew their flock's scab status and when appropriate, dipped properly, highlighting a need for greater cooperation in scab control. Farmers suggested increased awareness of scab and coordinated efforts could help create a cultural change to improve scab control.

## DISCUSSION

This pilot project was successful in establishing the farmer helpline and enrolling in excess of the target number of flocks for on‐farm clinical investigation. Sheep scab cases were identified in all counties of Northern Ireland (Figure 1) and at levels higher than previously reported, including among flocks sharing common grazing.^4^

Our findings highlighted barriers to managing scab, including negative skin scrapes from affected sheep as well as residual infestation following treatment, which, if not addressed, will hinder scab control. The serological test demonstrated how clinically suspicious sheep, with a negative skin‐scrape, could have hidden scab infestation identified, allowing prompt treatment and differentiating them from other sheep showing clinical signs not caused by scab, thereby preventing unnecessary treatments with an acaricide.^7^, ^14^ The project demonstrated the value of serology in detecting scab in flocks that are not showing clinical signs; particularly in flocks associated with the common grazing. These farmers were convinced that their flocks were unaffected, as would have been neighbouring farmers, or that purchasers had the sheep been offered for sale.

One central facet of the final push for scab eradication in Great Britain in the 1950s was convincing farmers of the presence of scab in their latently infested flocks, which often showed little or no clinical signs.^15^ Our results suggest that the same lessons around locally co‐developed solutions, supported by national co‐ordination and resourcing, need to be learned again, as has been borne out in sheep scab projects in England,^8^, ^16^ Wales^7^ and the Western Isles of Scotland.^17^

Farmers appreciated the role their vet played in the project, although some farmers in Northern Ireland have trouble accessing vets for flock health services,^18^ with similar reports from Scotland.^19^ Delays in the return of serology results hindered timely treatment on occasion. While veterinary visits and treatment were delivered within 14 days in many cases, there were concerning delays in the treatment of some flocks (Figure 2), risking local spread, as reported elsewhere^20^ and by farmers here. Future control programmes will need to understand the reasons for the delays observed between enrolment and treatment, which potentially include the farmer's ability to gather their flock or the availability of vets at times that suit sheep farmers. If the number of contiguous sheep‐grazing farms and flocks with access to common grazing areas is taken into consideration, veterinary manpower and logistics to expedite diagnosis and treatment will be needed^21^ to ensure that all contiguous flocks are gathered, tested and treated in a timely and coordinated manner.

Prevention of scab entering flocks will be paramount to greater scab control.^1^, ^5^, ^21^ Identified behaviours, including the purchase of replacement livestock in markets and suboptimal quarantine arrangements pose a risk to good scab control.^20^, ^22^ While it was clear that some of the participating farmers were spending time and money on preventative treatments,^23^ the approach taken was often unsuccessful, as scab outbreaks were still encountered.

Farmers who had scab confirmed in their flock believed that sheep movements were the most likely route by which the scab had entered their flock. However, nearly half (46%) of these farmers, during the follow‐up survey, still reported relying on visual observation, rather than a quarantine treatment, to prevent scab when introducing sheep to their flocks, with one in five reporting a quarantine period of less than 3 weeks. A minority (*n* = 8, 15%) failed to describe having any quarantine plan in place. This highlights the importance of further research to better understand the barriers to prevention, such as the practicality and cost of dipping small numbers of sheep, farmers’ experience in implementing more proactive scab prevention strategies^19^ and dipping prior to leaving markets^5^, ^24^ to break the transmission chain.

Of particular concern was the level of doramectin used as a preventative treatment, often followed by a scab outbreak. This injectable product has the benefit of being a single intramuscular administration.^25^ However, as there is insufficient persistence of effect, it cannot prevent scab from entering a flock. At best, where an undiagnosed incursion has occurred, it may, if all sheep are injected appropriately for their body mass and all moved to suitable, clean pasture or housing, eradicate scab from the flock before clinical signs occur. Of greatest concern is the potential to drive resistance to doramectin in both intestinal parasites and the sheep scab mite.^26^, ^27^ Prescribers should be urged to consult, in detail, about the clinical needs, expectations and requirements of farmers seeking a preventative scab treatment, particularly when asking for an ML. These results and previous work highlighted the importance of ensuring that an appropriate risk assessment is undertaken to target optimal preventative treatments and question whether preventative treatments are to be recommended at all, except in very high‐risk situations, such as common grazing.^23^

It was outside the scope of the work to determine the presence of clinical resistance within the mite population or whether the incorrect use of MLs led to the therapeutic treatment failures identified or the outbreaks of scab following the use of an ML as a preventive. Some farmers reported that prior to their involvement in the current project, they had to use a second, or in one case a third ML treatment, often ultimately dipping the flock, before they considered scab to be eradicated from their flock during outbreaks. This failure to clear infestation from flocks following an injectable ML treatment, both as reported in the RAFs and identified, using serology during the current project, raises concerns about the dissemination of scab from ML‐treated flocks where they are not subject to follow‐up testing. In Northern Ireland, the lifting of scab‐related movement restrictions only requires notification to the state authorities (DAERA), by a vet, that an authorised treatment has been administered.^3^ Thus, sheep with live mites could easily remain undetected if appropriately timed follow‐up blood testing is not legislated for and enforced.

Negative farmer perceptions hindered recruitment to the project, as evidenced by some potential participants declining a free diagnostic visit from their vet, alongside subsided flock treatment, worth up to £500 per flock despite describing classic clinical signs within their flock. The strained relationship between farmers in Northern Ireland and the state authorities has been previously documented in relation to scab control and other disease control programmes^4^, ^10^, ^28^ and may in part explain this reluctance. State support is crucial to progressing scab control in Northern Ireland. Only the government holds livestock movement data, which are essential for understanding risks associated with transportation of sheep previously highlighted as a significant cause of scab dissemination at the national level.^5^ These data are a necessary pre‐requisite to undertake movement tracings of animals onto and off farms where scab is diagnosed to permit risk‐based assessments to identify which farms are most likely to have infestations, enabling limited manpower and resources to be focused on the highest risk farms.^23^, ^29^ State authorities have the statutory powers to enforce the treatment of flocks, which, as farmers noted throughout the project can occasionally be necessary to ensure compliance.^20^

Some farmers, particularly in the follow‐up survey, recognised the need for a new approach with local coordination of diagnosis and treatment. They saw the benefits of access to a trusted and knowledgeable intermediary between the state and farmers to help farmers understand the implications of restrictions and to minimise the need for state officials to visit affected farms. This approach aligns with previous studies indicating that facilitation, coordination and targeting are more financially effective uses of resources and minimise the environmental and human health risks that the widespread use of dip would entail^7^, ^16^, ^23^ but requires some form of central funding, management and training support.

This pilot study was not designed to estimate the incidence or prevalence of scab infestation within Northern Ireland. The project case numbers were substantially higher than the one to two cases identified annually by state authorities and reported.^4^ Unfortunately, the inability to undertake tracing of sheep movements or routinely undertake testing on contiguous farms means that sheep with scab will have been left undiagnosed. For long‐term control of scab, there will be a need for local, or targeted, sharing of flock scab status data to ensure that all at‐risk flocks are investigated and treated. Delays between reporting suspicion, diagnosis and treatment, the use of inappropriate treatments and treatment failures combine to increase the risk of dissemination of *P. ovis* between and within flocks through animal movements. It is the energy and unanimity of farmers in their call for further, national control that is critical to future success and should urgently be supported.

## AUTHOR CONTRIBUTIONS

Paul Crawford identified the research need and developed the partnership to develop the project. Paul Crawford, Sam Strain, Stewart Burgess, Adewale Henry Adenuga and Aurélie Aubry sought funding and initiated the programme. Sharon Verner managed the data collection and initial analysis. Paul Crawford and Stewart Burgess reviewed and advised on blood test results. Paul Crawford wrote the first draft of the manuscript, and all the authors contributed to its critical review and refinement.

## CONFLICTS OF INTEREST

The authors declare they have no conflicts of interest.

## ETHICS STATEMENT

The authors confirm that the ethical policies of the journal, as noted on the journal's author guidelines page, have been adhered to. No ethical approval was required because the data were collected as part of normal veterinary practice.

## Supporting information



Supporting Information

## Data Availability

The consents obtained from participants does not permit the open dissemination of the data; however, all reasonable requests from researchers will be facilitated in providing further information from the primary data.
