# Supplementary material for: A study of sheep scab in Northern Ireland including detection and identifying barriers to control
Source: Vet Rec Open. 2024 Dec 23;11(2):e70003. doi: 10.1002/vro2.70003 (PMC11666421; doi:10.1002/vro2.70003)
Supplement: Supplementary file 1 — Supporting Information [file VRO2-11-e70003-s001.pdf]

Supporting Information

S1 Veterinary visit risk assessment form (RAF) and guidance

FARMER NAME
Address 1
Address 2
Postcode

Nominated Veterinary Practice:
AN Other Vets

Phone Number: 07123 123456

DATE

To whomever it may concern,

WHY HAVE I RECEIVED THIS LETTER?

Your practice has been the nominated by FARMER NAME who is participating in the Sheep Scab Project 'Stamp Out Scab,' to carry out a risk assessment for sheep scab, undertake an infestation investigation if necessary and provide advice on treatment if deemed to be required.

The farmer's individual reference number is: ---. This reference number must be used in all correspondence such as when submitting samples for testing to Biobest Labs.

WHAT IS THE PROJECT ABOUT?
Scab is a highly contagious disease which can lead to itching, reduced animal performance, wool loss and poor animal welfare. To date there has been little research undertaken on Sheep Scab within NI. This project has been designed to address this and will provide important information as the industry develops future strategies to control this disease. We hope to include around 100 flocks in the project.

This one-year project is funded through the Biotechnology and Biological Sciences Research Council.

WHAT IS THE REQUIREMENT OF THE PRACTICE?
Your veterinary practice is asked to contact the farmer to organise an on-farm visit at a time and date that suits both parties. Prior to visiting the farm contact your client and discuss why they are concerned about sheep scab in their flock. If there are no clinical signs but there has been a recent high-risk contact, it is advised to wait until at least 2 weeks have elapsed since the high-risk contact occurred to ensure that detectable levels of antibody will be circulating in the flock prior to any blood sampling.

During the visit, a veterinary surgeon from your practice is asked to examine the flock to investigate whether sheep scab is present, complete an infection risk assessment which you will receive, and, where necessary, collect samples for diagnosis. This is also an opportunity for the flock keeper to discuss any other flock health concerns they might have with you.

Testing for sheep scab can be by skin scrape to look for the scab mite under a microscope or by a blood test carried out by Biobest Labs. If blood tests are selected, 12 sheep should be sampled from each group of in contact animals. Blood test results should be available within two weeks.

IS THERE PROJECT SUPPORT?
The project is designed to support up to 100 flocks that have suspicion of sheep scab. Funding is available to cover the costs of a veterinary visit up to a maximum of £--- including VAT as well as the costs of blood test diagnostics. Invoices for farm visits carried should be sent to:

OFFICE ADDRESS; OFFFICE EMAIL
You must ensure that all paperwork is completed. This includes the risk assessment and the sample submission form where blood samples are taken. It is essential that all paperwork is returned to AHWNI completed accurately. Payment will only be made where all relevant paperwork has been fully completed.

Where treatment is advised the costs of medicine will be covered by the project up to a reasonable limit. For example, in general, it is expected that in most cases plunge dipping will be the treatment of choice. In this case the costs of dip will be covered by the project up to a reasonable limit. Given the typical size of NI flocks, it is likely that in most cases all the dip required for flock treatment will be paid for by the project.

IF SCAB IS PRESENT, WHAT WILL THE TREATMENT ENTAIL?
Dependent on the risk assessment, the flock and availability of resources you should provide advice on what form of treatment will result in the best outcome for this flock.
The two forms of treatment are:
1. Plunge dipper
2. Injectable products

NOTE: SPRAYS OR JETTERS ARE INELIGBLE TREATMENT OPTIONS.

In general, we advise the use of plunge dipping as the preferred treatment option given the lack of resistance that has been found to OP dips. The project can provide contact details for mobile dippers if the farmer does not have a preferred contractor.

Plunge dipping must fully comply with datasheet requirements and may be subject to unannounced audit during dipping. The project may be able to provide contact with a licenced dipper to use on farm facilities if they are of a suitable and safe standard where the farmer does not have a certificate. A holder of a certificate of competency must be in attendance during the dipping of the sheep.

Smaller flocks may want to consider transporting sheep a short distance if a dip can be organised locally.

Where dipping is to be considered ensure no recent (within 2 weeks of anticipated dipping date) OP or levamisole-based treatments have been given to the flock. Consider taking a snapshot of the medicine record book as evidence that you asked about recent treatments. Farmers should be advised that all routine flock management activities due in the coming weeks, should be undertaken before dipping or postponed until 3 or more weeks post dipping.

It may be advisable for farmers who do not have a license to start the process to obtain a licence for dip disposal if they do not already hold such and suggest they have secure temporary storage available on the day of dipping.

Where genuine reasons exist to avoid the use of OP dip, e.g., pre-existing medical condition or where they have been advised to avoid OPs the use of injectables may be considered. If this treatment option is chosen, consider if animal weights can be accurately measured to ensure accurate dosing and that it is likely that all sheep can be accurately dosed.

Once treatment has been applied it is crucial that treatment details are entered into the farmers medicine book.

WHAT HAPPENS IF SCAB IS SUSPECTED?
Sheep Scab is notifiable under the Sheep Scab (Northern Ireland) Order 1970 and any suspicious signs should be reported to your local DAERA Direct regional office. If sheep scab is suspected you are required to inform DAERA of this and DAERA will then apply movement restrictions on the flock.

DAERA restriction scenarios:

1. Where there is an initial notification of suspicion of sheep scab in a flock, a SS2 notice will be issued by DAERA which restricts movement of sheep out of the flock. A movement licence to an abattoir for slaughter can be used but these must be obtained from the flock keeper's local DVO office.
2. If on clinical examination no evidence of sheep scab in the flock is identified a SS3 notice will be issued by DAERA lifting any flock restriction relating to sheep scab.
3. If sheep scab is confirmed in a flock (skin scrape/lab positive disease confirmation) a SS4 notice will be issued by DAERA which restricts movement of sheep both into and out of the flock. A movement licence to an abattoir for slaughter can be used but these must be obtained from flock keeper's local DVO office.
4. Once treatment is confirmed to have taken place a SS3 notice will be issued by DAERA lifting any flock restriction relating to sheep scab.

DAERA have been informed of this project and no DAERA official will be required to visit any flocks because of sheep scab being suspected.

HOW WILL RESTRICTIONS BE LIFTED?
Vets are required to inform DAERA that a negative result has been received or the date that treatment occurred. This is to allow DAERA to issue the farmer with a notice that restrictions are lifted. The date of lifting restrictions will vary dependent on what product is used.

WHAT WE NEED FROM YOU?

In order to receive payment AHWNI require the veterinary practice to send completed on-farm questionnaires, as well as inform AHWNI of the outcome of blood test results, when DAERA has been informed that scab is suspected and when treatment has happened.
On the invoice practices MUST QUOTE THE FARMER'S UNIQUE FARM IDENTIFIER NUMBER.

For more information please contact AHWNI:
Email: SCHEME CONTACT EMAIL

Risk assessment data capture form for completion by veterinary surgeon during farm visit

Form A: Initial Farm Visit (one form per business) – Visit Date: ___/___/___

**Farm unique identifier**		
**Contact Name**		
**Farmer's business name and address**		
**Email address and telephone contact**		
**Flock number**		
**Farm vet details**		
**Farm location**	____ (Postcode or OS coordinates)	
**Farm details**	Grazing height above sea level: __________________ m/ft (delete one)<br><br>Overall farm area: ________________ acres/ha (delete one)<br><br>Sheep only ☐, mixed livestock ☐, livestock/arable ☐ (tick one)	
**Flock details – please provide numbers**	Breeding ewes | | Rams | | Other sheep |	
**Sheep location** *i.e. are the sheep run separately or together. If the farm is in geographically distinct areas or if they graze land away from the farm, do sheep grazing different areas mix and for how long?*	Are all animals run as a single flock? Yes ☐ No ☐<br>If not, please provide details:	
**Lambing details**	Lambing date(s): circle month(s)<br><br>Jan | Feb | Mar | Apr | May | Jun | Jul | Aug | Sept | Oct | Nov | Dec<br><br>Any major management changes in last 3 years: Yes ☐ No ☐<br>If yes, then give details:	

Replacement policy (tick all that apply)

	Homebred	Bought in	If bought in please give details e.g. market, direct from farms etc.
Ewes			
Rams			
Others			

Grazing details

For common grazing, indicate times that sheep are on the commons

Jan	Feb	Mar	Apr	May	Jun	Jul	Aug	Sept	Oct	Nov	Dec

What proportion of the flock are grazed on the commons? _______________

Do sheep grazed on the common mix with other sheep in the home flock? ___________

Are any of the sheep wintered away? Yes ☐ No ☐ Part of flock only ☐
Details:

If yes, when do they go and come back?

Sep	Oct	Nov	Dec	Jan	Feb	Mar	Apr	May

Scab history

Does the farmer currently have or suspect scab in their flock? Yes ☐ No ☐

Have they previously had scab in their flock? Yes ☐ No ☐

If yes, in which of the last 10 years have they had scab?

2013	2014	2015	2016	2017	2018	2019	2020	2021	2022

Was the diagnosis confirmed by a vet? Yes ☐ No ☐

When they last had scab, how and when did they treat it?

	**Active Ingredient**	**Product name** (application method)	Tick any used	Date of last treatment (DD/MM/YYYY)
OP via plunge dip	Diazinon			
OP via shower or jetter	Diazinon			
Injection	ML			

Neighbouring farms: (contiguous farms or those sharing grazing, including all outfarms and other conacre or winter grazing)

How many neighbours have sheep flocks (consider all the ground that has been used to graze sheep over in the past 6 months)	

Have any of the farm’s immediate neighbours had scab in the last 10 years?

Yes ☐ No ☐☐Don’t know ☐

If yes, when? Please circle:

2013	2014	2015	2016	2017	2018	2019	2020	2021	2022

Are there any particular boundaries that have a regular problem with scab?
Yes ☐ No ☐☐Rather not comment ☐

If yes, please give details of boundaries that are the most risky for scab:

<table>
<tr><td></td><td></td></tr>
<tr><td>Current strategy against scab</td><td>In the last 12 months has a preventative treatment against scab been used?
Yes ☐ No ☐☐If yes – when?

Jan	Feb	Mar	Apr	May	Jun	Jul	Aug	Sept	Oct	Nov	Dec

What product(s) were used?

	**Active Ingredient**	**Product name** (application method)	Tick any used
OP via plunge dip	Diazinon		
OP via shower or jetter	Diazinon		
ML	Injection		

If no preventative treatments are used, does the farmer only treat when they see signs of scab?
Yes ☐ No ☐

How do they normally diagnose/decide they have scab in the flock?
☐ Recognise signs based on experience
☐ Vet wool exam or skin scrape
☐ Blood test (ELISA)
☐ Assume based on scab in neighbouring flocks

Any other strategies used: ____

If they currently use OP dip:		
Do they have their own dipping facilities?	Yes ☐	No ☐
Do they have a Safe Use of Sheep Dip certificate?	Yes ☐	No ☐
Do they have a licence to dispose of used dip?	Yes ☐	No ☐
Do they use a mobile sheep dip contractor?	Yes ☐	No ☐
If they don't currently used OP dip, would they be willing to do so in the future? If no, why not?	Yes ☐	No ☐
Are they willing to coordinate treatments and timings with their neighbours? If no, can they say why not?	Yes ☐	No ☐
Have they used OP to prevent blowfly strike this year? If so please indicate during which month(s) below:	Yes ☐	No ☐
</td></tr>
<tr><td></td><td>

Jan	Feb	Mar	Apr	May	Jun	Jul	Aug	Sept	Oct	Nov	Dec
</td></tr>
</table>

Quarantine / biosecurity

Are <u>all</u> bought in (or returning) animals yarded/quarantined on arrival
Yes ☐ No ☐

If yes, for how long are they kept separate from the rest of the flock?
____

Are there any differences in quarantine policy for ewes, rams, store lambs etc.?

Does the flock owner ask for and receive information from the sellers about treatments for scab given to any bought-in animals? Yes ☐ No ☐

Do they give any treatments for scab to bought-in animals, irrespective of the information received from the seller: Yes ☐ No ☐

Where do they suspect the sheep scab now in their flock came from?

Impact on animal performance

Which key performance indicators does the flock owner feel have been most impacted by sheep scab in their flock (tick up to 4)?

(a) Lambing percentage/ ewe prolificacy ☐
(b) Lamb mortality ☐
(c) Lamb growth rate up to weaning ☐
(d) Lamb growth rate after weaning ☐
(e) Ewe live weight and body condition score ☐
(f) Ewe mortality ☐
(g) Ewe barren rate ☐
(h) Ewe culling rate ☐
(i) Other (please specify) ☐________________
(j) No Impact on flock performance ☐

In your veterinary opinion, what is the average severity of the sheep scab in the flock?

**Tick (ü)**	**Severity level**	**Symptoms may include**
	No symptoms seen	None visible
	Mild symptoms seen	Some restlessness, rubbing against fence posts, soiled and stained areas of wool, head tossing and/or loose tags of fleece
	Moderate symptoms seen	Moderate restlessness, rubbing against fence posts, soiled and stained areas of wool, head tossing and/or loose tags of fleece
	Severe symptoms seen	As above but with noticeable areas of wool loss, clumping or clotting of wool, damaged moist red skin and/or dry crusty scabs

VET CHECKLIST

1. **Complete Form A Initial Farm Visit**
2. **Complete Form B Blood Sample**
3. **Skin scrapes taken from affected sheep**
4. **Blood samples (x12) taken for dispatch to Biobest Laboratories**

Are there clinical signs of Sheep Scab in the flock today? **Yes ☐ No ☐**

5. If Sheep Scab is suspected, or confirmed by microscopy, send a Notification form to your local DAERA office by e-mail immediately, copying AHWNI <u>info@animalhealthni.com</u> into the e-mail.
DAERA e-mail addresses are listed on the Notification form.

Signed: ________________________________

Name: ________________________________

Date of visit: ________________________________

S2 End of project feedback survey questions.

The following questions were delivered via the Google Forms application to all farmers who agreed to participate in the project.

1. What was the outcome of testing your flock in the project?
    - NEGATIVE for scab
    - POSITIVE diagnosis of scab, by skin scrape only
    - POSITIVE diagnosis of scab, by blood sample only
    - POSITIVE diagnosis of scab, by skin scrape AND blood sample
    - Advise to MONITOR the flock
    - SUSPICION of scab

2. If scab was detected in your flock, to what extent did it affect the performance of your flock?
    - Not at all
    - Small extent
    - Moderate extent
    - Severe extent
    - N/A

3. If you used mobile dipping for treatment of scab or as a preventative measure before use of common grazing, how satisfied were you? From 1, 'very unsatisfied' to 5, 'very satisfied.'
    1 2 3 4 5

4. Do you use common grazing?
    - Yes
    - No
    - Sometimes but not at present

5. Would you be willing to coordinate the timing of scab treatments with your neighbours in future?
    - Yes
    - No
    - Not sure

6. Regarding communications, the veterinary visit, testing for scab, flock restriction/ derestriction and treatment, what aspects of the project delivery worked well and what could be improved?
    (Free text responses)

7. How do you plan to control sheep scab in future in your flock?
    - Blood test and dip if Positive
    - Use injectable without testing
    - Not sure
    - Use dip without testing
    - Blood test and inject if Positive

8. How do you deal with sheep that are being introduced to the flock? (Tick all that apply.) (51 respondents)
    - Dip on entry
    - Treat with an injectable for scab
    - Quarantine for more than 3 weeks
    - Quarantine for less than 3 weeks
    - Separate yarding

9. How has the project affected the control of scab in your area?
    (Free text responses)

10. Do you think that a programme dedicated to the control of sheep scab in NI would be useful in the future and if so how should this be funded?
    - Not useful
    - Useful, fund through NI Assembly / DAERA
    - Useful, fund through industry contributions
    - Useful, fund through shared government and industry contributions

11. What could industry do better or differently if working on a national control programme for sheep scab?
    (Free text responses)

S3 Additional exemplar quotes from follow-up survey respondents

Follow-up survey

What can the wider industry do?

SR09: Make it compulsory to plunge dip sheep annually

SG39: If vets at the marts were more careful and not afraid to report, I think it would help. I believe they have been known to turn a blind eye to such a situation. Because it's not clamped down on, farmers who are suspicious just sell affected sheep to get out of the risk of being closed down or reported.

SR25: Random testing on farm and in markets.

SR17: Veterinary inspections and testing at the unloading bays of livestock marts is needed. Without this it is too easy for a farmer to sell infected sheep to get rid of his or her problem. This is not doing any good for the national control of scab. The irresponsible farmer will always be the weakest link

SR03: There should be a national fencing programme. I am constantly fencing out my neighbours' livestock. They refuse to do any fencing themselves, as they claim they can't afford it.

SR14: All flocks should be compulsory dipped. More courses should be made available for the use of dip on farms, including help with disposal of the waste products.

What the project did well or could do better

SR06: Think that something similar to the sheep scab project would be very helpful if rolled out on a national level.

SR28: Everything went to plan. Need to ensure everyone using common grazing dips.

SR44: All worked well but it makes you feel like bad stockmanship when flock came up positive

SR36: I was happy with all aspects especially being able to say when it suited me to test.

SR45: Quicker return of test results would be good.

SR17: The flock restriction and derestriction were a total letdown for me, and for this reason alone I would not be willing to recommend the scheme to anyone

SR53: I rang the state authorities to tell them about outbreak. They didn't close me down. However, they tried to close me down after treatment was finished.

SR31: All sheep in neighbourhood dipped at the same time

SR19: The results were a bit long in coming. Treatment worked well. Communication regarding getting flock derestricted was not good.

SR50: Worked well: the in-person workshop, the friendly non-judgemental advice on phone, the follow up calls, allocating dip concentrate to contract dippers, supported vet visit and skin testing. Less well: relying on assumption that other commonage users had dipped and reluctance of Common's trustees to help the process.
